# Synthesis and Properties of Bulk Mg_3_WN_4_ in a Wurtzite-Derived Structure

**DOI:** 10.1021/acs.chemmater.6c00200

**Published:** 2026-06-24

**Authors:** Anna A. Berseneva, Christopher L. Rom, Layton Rudolph, Yunseung Kuk, P. Shiv Halasyamani, Rebecca W. Smaha, James R. Neilson, Andriy Zakutayev

**Affiliations:** † Materials Science Center, 53405 National Laboratory of the Rockies, Golden, Colorado 80401, United States; ‡ Department of Chemistry, 3447 Colorado State University, Fort Collins, Colorado 80523, United States; § Department of Chemistry, 14743 University of Houston, Houston, Texas 77204, United States; ∥ Department of Chemistry and School of Materials Science & Engineering, Colorado State University, Fort Collins, Colorado 80523, United States

## Abstract

Experimental synthesis of theoretically predicted materials with controlled elemental coordination environments can lead to the realization of useful properties, such as facile ion transport or ferroelectric switching. Among such materials are ternary nitrides in the Mg−W−N composition space, where several stable and metastable compounds have been predicted and synthesized recently in bulk and film forms. Here, we report for the first time on the bulk synthesis of Mg_3_WN_4_ in a wurtzite-derived crystal structure via a solid-state metathesis reaction. *In situ* synchrotron powder X-ray diffraction shows how the ion exchange proceeds from Li_6_WN_4_ + MgCl_2_ precursors to Mg_3_WN_4_ + LiCl products, with the reaction starting slowly near 380 °C and completing by 600 °C, including the presence of a competing disordered rocksalt-derived phase (Mg,W)N above 440 °C. The follow-up *ex situ* powder synthesis at 400 °C for 0.5 h with 10% excess MgCl_2_ reveals the cation-ordered nature of the wurtzite-derived Mg_3_WN_4_ structure with polar symmetry confirmed by second-harmonic generation measurements. Optical absorption spectra, chemical composition analysis, and electron microscopy imaging suggest that bulk Mg_3_WN_4_ obtained via metathesis reaction is prone to defect formation. Overall, this study shows that selective *ex situ* synthesis of the phase-pure ternary nitrides, informed by *in situ* measurements, is possible by carefully controlling the thermal budget of the reaction and paves the way toward property characterization of the Mg_3_WN_4_ wurtzite-derived material.

## Graphical abstract

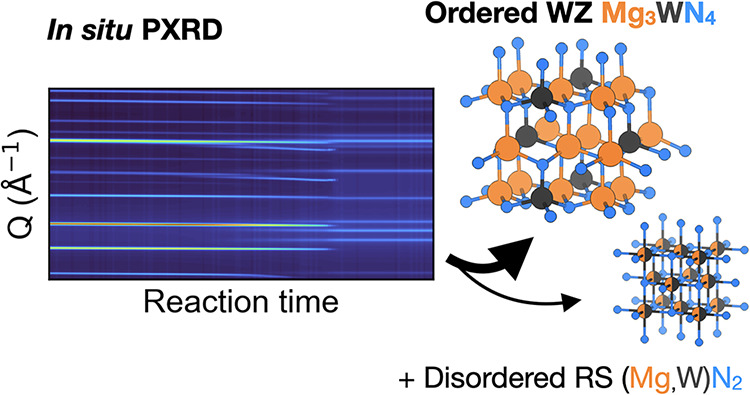

## Introduction

Controlling the local elemental coordination environments often leads to beneficial material properties. For example, tetrahedrally coordinated Mg^2+^ is more predisposed toward Mg^2+^ ionic mobility than Mg^2+^ in octahedral environments, according to a prior theoretical study.^1,2^ However, materials with Mg^2+^ in a pure tetrahedral environment are difficult to find, since Mg^2+^ in octahedral coordination is more common. Another theoretical study^3^ aimed at the discovery of tetrahedrally bonded wurtzite ferroelectric materials beyond (Al,Sc)­N^4^ predicted that Mg^2+^ ions in tetrahedral coordination are promising local structural features for facile ferroelectric switching in nitrides with wurtzite-derived (WZ) crystal structures, compared to much more common tetrahedrally coordinated Zn^2+^ atoms with lower bond ionicities. This makes the hypothetical Mg_3_WN_4_ material a more promising ferroelectric than the previously synthesized Zn_3_WN_4_.^5^ An additional interesting aspect of the hypothetical Mg_3_WN_4_ is that its reported Mg_3_MoN_4_ cousin shows a collective rather than individual switching pathway, unlike many other wurtzite nitride ferroelectrics.^3^ Thus, the tetrahedrally coordinated Mg^2+^ local structural feature in WZ Mg_3_WN_4_ makes this material interesting as a potential solid-state Mg^2+^ ion conductor and as a promising ferroelectric material.

A large computational and experimental study predicted numerous ternary nitrides and experimentally realized several of them.^6^ Among them, Mg_3_WN_4_ and Zn_3_WN_4_ were predicted in a WZ structure type (NLR MatDB ID 290104) with tetrahedrally coordinated Mg^2+^ and Zn^2+^ cations, respectively.^7^ According to theoretical calculations, WZ Zn_3_WN_4_ and Mg_3_WN_4_ adopt a polar structure (*Pmn*2_1_), with large calculated band gaps (3.96 and 5.17 eV, respectively) based on the GW method.^8,9^ As a part of that study, we synthesized Zn_3_WN_4_ in a cation-disordered WZ structure via thin film sputtering,^6^ with an optical absorption onset around 2 eV. Subsequently, we reported a cation-ordered polymorph of Zn_3_WN_4_ that exhibited the 2 eV optical absorption onset.^5^ However, experimental synthesis of Mg_3_WN_4_ in a WZ crystal structure has not been reported, and only the rocksalt crystal structure is known at this composition.^10^

Our prior experimental work focusing on the Mg−W−N chemical space realized several compounds in various structure types.^10^ We discovered MgWN_2_ in a layered ’rockseline’ (RL) structure using ceramic synthesis techniques and used sputtering to yield thin films of Mg_*x*_W_1−*x*_N in cation-disordered rocksalt (RS; 0.1 < *x* < 0.9) and hexagonal boron nitride (h-BN; 0.7 < *x* < 0.9) structure types (Figures 1 and S4). Annealing thin films of *x* ∼0.5 was used to convert the disordered RS structures to cation-ordered RL structures for MgWN_2_,^11^ but for the *x* ∼0.75 composition, Mg_3_WN_4_ remained in the RS structure up until the decomposition temperature of 1000 °C.^10^ Combined with the density functional theory calculations, these experiments showed that RL MgWN_2_ defined the convex hull of the Mg−W−N chemical system. Cation-ordered WZ Mg_3_WN_4_ is therefore predicted to be enthalpically metastable at 0 K (+0.063 eV per atom above the hull).^10^ As a result, the synthesis of WZ Mg_3_WN_4_ has eluded us so far.

**Figure 1:**
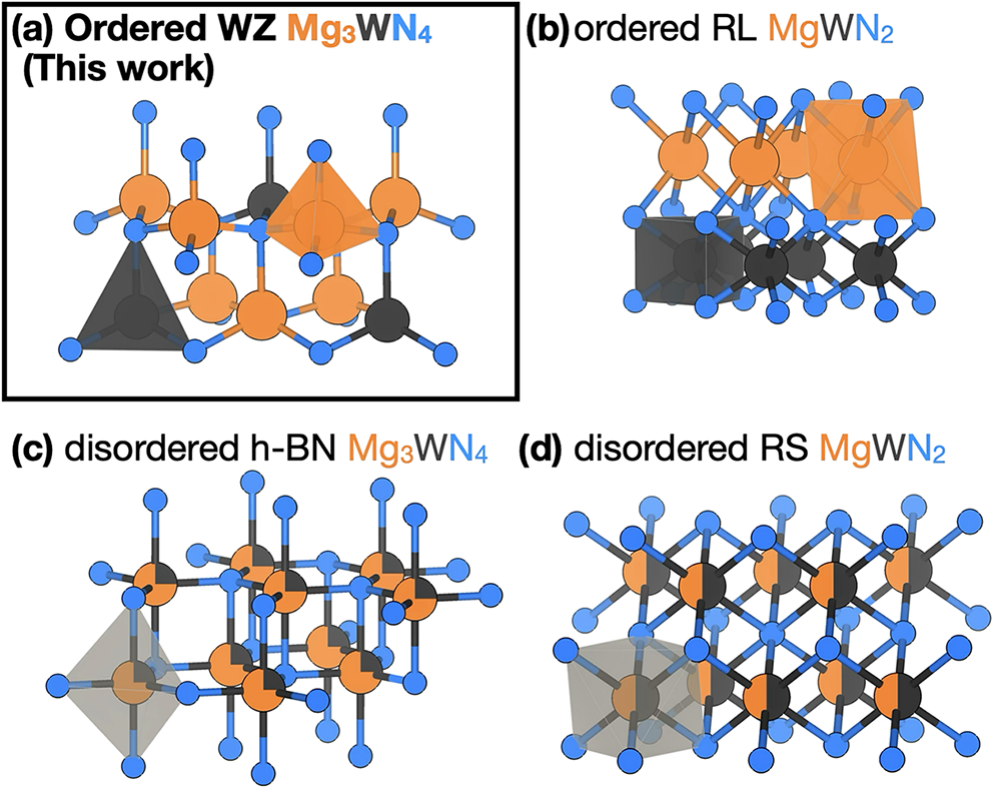
Several structures form in the Mg−W−N phase space including (a) cation-ordered, WZ Mg_3_WN_4_ (described here), and previously reported (b) rockseline, (c) h-BN, and (d) rocksalt phases.

More recently, ion exchange approaches have been used to make cation-ordered versions of other ternary nitrides, including metastable structures. Starting from a ternary Li_2_ZrN_2_ precursor, metastable polymorphs of MgZrN_2_ and MgHfN_2_ with cation-layered structures (space group: *R*3̅*m*) have been synthesized.^12^ This metastable layered structure contrasts with the calculated ground-state cation-ordered structure (*I*4_1_/*amd*) predicted by theory^6^ and with the previously synthesized cation-disordered structure of MgZrN_2_.^13–15^ During this cation-exchange reaction, 2Li^+^ and Mg^2+^ undergo ion exchange topochemically, preserving the layers of octahedral [ZrN_6_] according to *in situ* synchrotron powder X-ray diffraction (PXRD) measurements. In the past, similar cation exchange reactions have been used to make other metastable layered nitrides, such as CuTaN_2_ ^16^ and CuNbN_2_.^17^ Together, these results indicate that ion exchange approaches may be suitable for experimentally realizing the theoretically predicted metastable Mg_3_WN_4_ with tetrahedral Mg^2+^ coordination and promising ferroelectric switching or Mg^2+^ ion transport properties.

Here, we report the synthesis of Mg_3_WN_4_ in a cation-ordered WZ structure (*Pmn*2_1_), as observed by *in situ* and verified by *ex situ* PXRD data. The Mg_3_WN_4_ material forms from the reaction of Li_6_WN_4_ and MgCl_2_ precursors in the range of 400−600 °C. The results of the *ex situ* PXRD measurements indicate that this cation-ordered WZ phase forms alongside a cation-disordered RS impurity. *In situ* synchrotron measurements show that the WZ Mg_3_WN_4_ phase begins forming at lower temperatures (ca. 380 °C) than the RS polymorph (ca. 440 °C). This knowledge lets us synthesize the phase-pure WZ Mg_3_WN_4_ compound at 400 °C for 0.5 h with 10% excess in the lab by washing away unreacted Li_6_WN_4_, which is still present at these lower temperatures. Obtaining a phase-pure product allows us to measure optical absorption spectra with diffuse reflectance (DR) spectroscopy, comparing Mg_3_WN_4_ to the Zn_3_WN_4_ analog, as well as to perform second-harmonic generation (SHG) measurements to support the polar space group (*Pmn*2_1_) of both these materials. This work, informed by *in situ* measurements, establishes the *ex situ* synthesizability of phase-pure Mg_3_WN_4_ in a cation-ordered wurtzite-derived structure, which is of significant interest for possible ferroelectric switching or Mg^2+^ ionic conductivity applications.

## Methods

### Synthesis

As some precursors are highly moisture-sensitive, all precursors were prepared and stored in an argon-filled glovebox (O_2_ < 0.1 ppm, H_2_O < 0.1 ppm) unless explicitly mentioned. Li_3_N (≥99.5%, 80 mesh, Sigma-Aldrich), W (99.95%, <1 μm powder, Thermofisher Scientific), and MgCl_2_ (Sigma-Aldrich, 99.99%, AnhydroBeads) were used as received.

Zn_3_WN_4_ was synthesized using the method from our previous report.^5^ Li_6_WN_4_ was synthesized using a method modified from the literature,^18^ as we described previously.^5^ Briefly, solid precursors (2.1 Li_3_N + W, where the ca. 5 mol % excess Li_3_N accounts for loss by evaporation) were ground with an agate mortar and pestle and loaded into custom Zr crucibles with Zr lids (ca. 1 g of loose powder) and then heated under flowing N_2_ (50 sccm, 99.999% purity) with a 5 °C/min ramp followed by a 12 h dwell at 850 °C. Samples were cooled by turning off the furnace and then recovered into the glovebox without air exposure. The air-free transfer of reagents and the final product was done with a tube equipped with the leak-free quick disconnect flange sets by the end of the tube. The resulting powders were beige in color and phase-pure by PXRD.

Metathesis reactions were performed by homogenizing 3MgCl_2_ + Li_6_WN_4_ with an agate mortar and pestle and pelletizing at ca. 1 ton pressure in a 6 mm die, loading the pellet into an alumina crucible, and heating without exposure to air, as described in the text. Postreaction, samples were recovered into the glovebox without air exposure. The byproduct LiCl was removed via washing with anhydrous methanol in a glovebox.

The purest sample was obtained via the reaction between 3.3MgCl_2_ and Li_6_WN_4_. Solid precursors were ground, pressed into a pellet, loaded into an alumina crucible, and then heated under flowing N_2_ (50 sccm, 99.999% purity) with a 10 °C/min ramp followed by 0.5 h dwell at 400 °C. The sample was cooled by turning off the furnace, recovered into the glovebox without air exposure, and then washed with anhydrous methanol.

### Compositional Analysis

Scanning electron microscopy (SEM) and energy-dispersive spectroscopy (EDS) were performed to visualize particles and semiquantitatively assess the composition. SEM imaging was done on a Hitachi S-4800 SEM operating at 15 keV accelerating voltage and 10 μA beam current. Elemental analysis was conducted by EDS on the same instrument using the included Pathfinder analysis software for quantification. EDS was performed directly on powder mounted on an SEM stub with carbon tape. Spectra were acquired for 60 s. EDS mapping was performed by acquiring data for 6 min. Note that C, Al, and Si are always present in the EDS spectra coming from carbon tape, the Al SEM stub, and the detector, respectively.

Wavelength dispersive X-ray fluorescence (WD-XRF) was performed to semiquantitatively assess the composition using a Rigaku ZSX PrimusIV. Data collection was performed on powder capped with the Etnom 3.0 μm thin film in the Ar glovebox. Note that the O element is always present in this setup coming from the Etnom film and that the N element cannot be determined in this configuration due to the full N Kα edge absorption by the film.

### *Ex Situ* Laboratory PXRD

The products of all reactions were characterized by powder X-ray diffraction (PXRD). Laboratory PXRD patterns were collected on a Rigaku Ultima IV diffractometer and a Rigaku SmartLab in Bragg−Brentano geometry with Cu Kα X-ray radiation at room temperature. For the Rigaku Ultima, we used 10 mm and 2/3° divergence slits, 0.6 mm and 2/3° receiving slits, 5° incident and receiving Soller slits, a K_β_ filter, and a 0.02° step with 0.5 h collection; several scans for the same samples were merged for the Rietveld refinement. For the Rigaku SmartLab, we used a 10 mm vertical divergence slit, 20 mm and open receiving slits, 2.5° incident and receiving Soller slits, a Kβ filter, and 0.001° step size with 1 h collection; the scan was collected with a variable width divergence slit; therefore, slit correction was applied in SmartLab Studio II software. All reaction products were initially prepared for PXRD measurements inside the glovebox. Powder was placed on off-axis cut silicon single-crystal wafers to reduce background scattering and then covered with polyimide tape to impede exposure to atmosphere. After Mg_3_WN_4_ was determined to be moderately air-stable, PXRD patterns of the washed Mg_3_WN_4_ were collected without polyimide tape to decrease the background signal. Rietveld refinements were performed using TOPAS-64 v6 ^19^ and GSAS-II.^20^ Lattice parameters, atom coordinates, microstrain broadening, and thermal parameters were refined for the purest WZ Mg_3_WN_4_ phase (*Pmn*2_1_, *R*_wp_ = 2.869%) and are presented in Table 1. For a stable refinement, *U*_iso_ parameters for N atoms were fixed at 0.01 Å^2^ and not allowed to refine; thermal parameters for the Mg and W sites were freely modeled, with the *U*_iso_ for Mg1, Mg2, and W1 constrained to be equivalent. We tried to refine *U*_iso_ values for Mg1, Mg2, and W1 separately, but this resulted in aphysical *U*_iso_ values for Mg1 and Mg2 (<0.0001 Å^2^). Free refinement of metal occupancies resulted in values close to 1 for W1, while Mg1 and Mg2 refined to 1.05(1) and 0.94(1), respectively. Modeling Mg and W disorder with equivalent *U*_iso_ values yielded *R*_wp_ values in the 2.84−2.87% range, which was an insignificant improvement taking into account the increase in parameters (from 21 to 24). Addition of an RS MgWN_2_ phase led to 4.3 wt % of RS phase but did not lead to significant model improvement: the *R*_wp_ refined to 2.660%, yet the parameter number increased from 21 to 24. Free metal occupancy refinement, disorder modeling, and addition of an RS phase insignificantly affected *R*_wp_, and therefore, none of these was used in the final model.

### *In Situ* Synchrotron PXRD

*In situ* synchrotron PXRD measurements were conducted at the beamline 17-BM-B of the Advanced Photon Source at the Argonne National Laboratory. For these experiments (λ = 0.25306 Å), the PerkinElmer plate detector was positioned 800 mm away from the sample. Homogenized precursors were packed into quartz capillaries in an Ar glovebox and flame-sealed under vacuum (<40 mTorr). Capillaries were loaded into a flow-cell apparatus^21^ and heated at 10 °C/min to the specified temperature. A thermocouple was placed against the tip of the sample capillary, approximately 2 mm horizontally from the position of the X-ray beam. Diffraction pattern images were collected every 30 s by summing 100 exposures of 0.1 s each (10 s of summed exposure), followed by 20 s deadtime. Images collected from the plate detector were radially integrated using GSAS-II and using a silicon standard.

Sequential Rietveld refinements were conducted on *in situ* synchrotron PXRD datasets using TOPAS-64 v6.^19^ Lattice parameters, background terms, and scale factors were refined for each phase as a function of temperature, while atomic coordinates and occupancies were held constant at the initial values of the reference structure. A weighted scale factor (WSF) was calculated for each phase p as a product of scale factor *S*, cell volume *V*, and cell mass *M*: (WSF)_p_ = *S*_p_ *V*_p_ *M*_p_.^22^ To derive a mole value for each phase, we used the (WSF)_p_/*M*_rp_ formula, where *M*_rp_ is the molar mass and normalized it for all phases with the initial value for Li_6_WN_4_ as 1 (Figures S2 and S3). Data presented in the mole scale factor support the inclusion of Mg into the LiCl phase and/or Li_2_MgCl_4_ formation. Owing to the substantial overlap between the RS (Li,Mg)Cl phase and Li_2_MgCl_4_, we were not able to reliably extract the WSF for both phases while allowing the unit cell size to refine. Instead, we represent the Li_2_MgCl_4_ phase in Figure 2 using the normalized peak intensity at *Q* = 1.026191 Å^−1^ as a proxy for its WSF. We note that amorphous and liquid phases are inherently not observed in powder diffraction measurements and therefore cannot be accurately included in this analysis. A Lorentzian size broadening term was refined for each phase to model the peak shape using the pattern showing the greatest intensity of the relevant phase; this term was then fixed for the sequential refinements to better account for changes in intensity. To improve reliability in the sequential refinement, isotropic displacement parameters (*B*_iso_) were fixed at 1 Å^2^ for all atoms, but we note that this is likely not physical for a variable temperature investigation.

**Figure 2:**
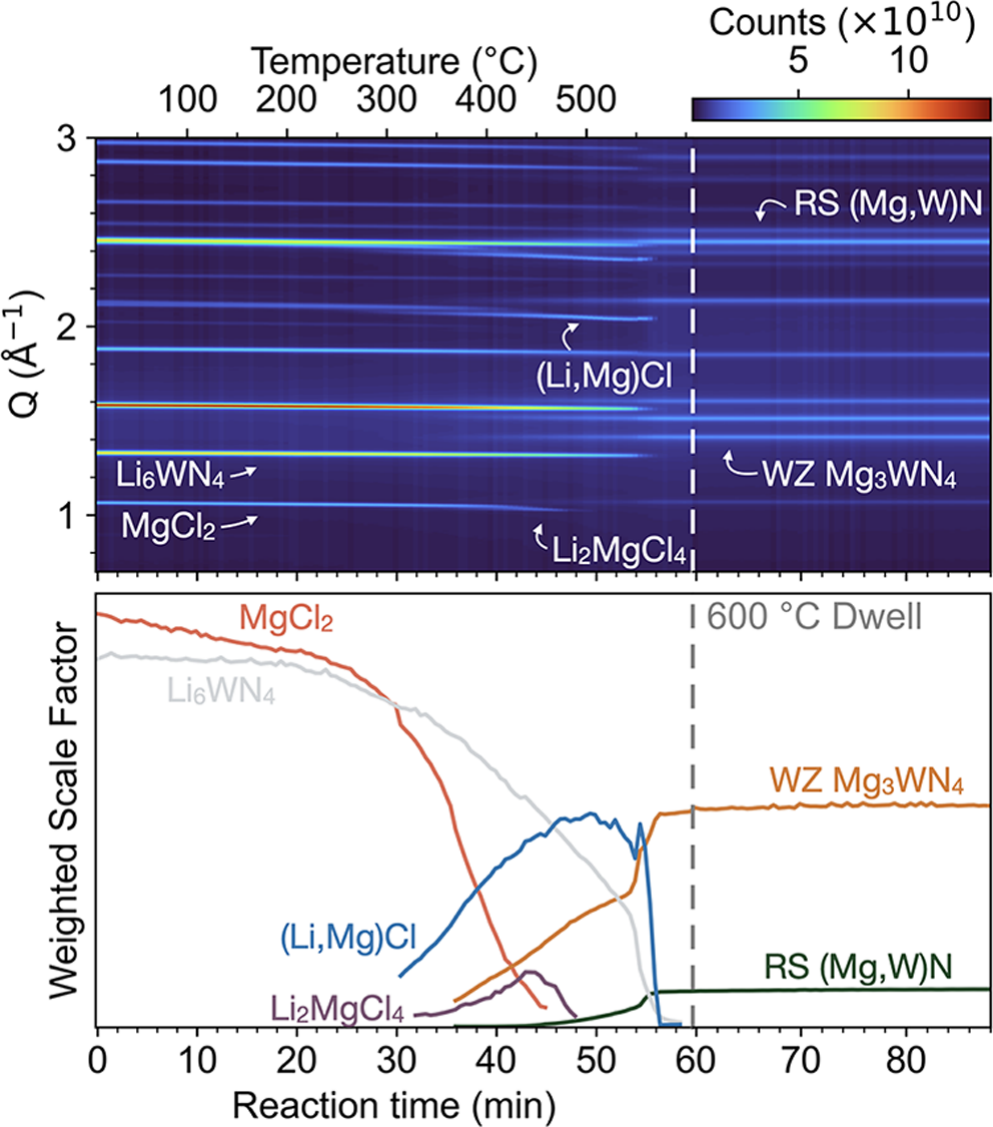
*In situ* synchrotron PXRD of the reaction between 3MgCl_2_ and Li_6_WN_4_, showing that the reactivity starts close to 400 °C and that the reaction is complete by 600 °C. We represent the Li_2_MgCl_4_ phase using the normalized peak intensity at *Q* = 1.026191 Å^−1^ as a proxy for the weighted scale factor (WSF).

### Optical Characterization

The absorption of the bulk Mg_3_WN_4_ and Zn_3_WN_4_ samples was measured using a Cary 7000 optical spectrophotometer with the sample mounted outside an integrating sphere, diffuse reflectance accessory (DRA) module, using a 1/4″-diameter aperture plate attached to the reflectance port. Ground powder samples were pressed in between two 1/16″-thick fused silica glass slides (1″ × 1″) from GM Quartz and fixed with epoxy applied around the perimeter of the slides. After baselining with the glass slides, the absorption spectra were collected in the 190−800 nm range with a reduced slit, with 120 nm/min rate and 1 nm step size. Relative absorption (*A*) was estimated as the Kubelka−Munk function, *A* = (1 − *R*)^2^/2*R* where *R* is the reflectance.

Powder second-harmonic generation (SHG) measurements were carried out using a modified Kurtz−Perry powder technique,^23^ employing an Nd:YAG laser operating at 1064 nm and a Ho:YAG laser operating at 2090 nm as fundamental light sources. KH_2_PO_4_ and AgGaS_2_ were used as reference materials for SHG measurements at 1064 nm and 2090 nm, respectively. Polycrystalline samples of Mg_3_WN_4_, Zn_3_WN_4_, and the reference materials were packed into sample holders. The SHG signals were collected using a photomultiplier tube and recorded with an oscilloscope.

## Results and Discussion

We initially synthesized Mg_3_WN_4_ powders via a metathesis reaction at ambient pressure in flowing nitrogen gas:

1 $${\mathrm{Li}}_{6}{\mathrm{WN}}_{4}+3{\mathrm{MgCl}}_{2}\to {\mathrm{Mg}}_{3}{\mathrm{WN}}_{4}+6\mathrm{LiCl}$$

The unoptimized reaction of heating at 600 °C for 12 h yields a black powder. Washing with anhydrous methanol removes the LiCl byproduct. The EDX in SEM quantifies atomic percentages as shown in Table S4, although the N content could not be determined precisely. The Mg/W ratio of ∼3.5−4 indicates that Mg_3_WN_4_ is the likely product. These composition data are derived from the EDS spectra collected from the three separate agglomerates of crystallites shown in Figure S12. Moreover, the cation composition of the bulk sample was confirmed by wavelength dispersive X-ray fluorescence (WD-XRF), matching well with the EDS results (Table S4).

*Ex situ* laboratory PXRD data on the washed reaction products show that the Mg_3_WN_4_ phase is the main product (close to 70 wt %), in the orthorhombic space group *Pmn*2_1_ (no. 31). This structure is derived from the polar wurtzite (WZ) crystal structure by ordering of Mg^2+^ and W^6+^ on the cation sublattice and hence is also polar.^24^ Other possible cation-ordered derivatives of the WZ structure including *P*31*c* (no. 159),^7^ or the potential nonpolar h-BN or BeO_2_ parent compounds, do not fit the PXRD data, as shown in Figure S4. In addition to the WZ phase, a substantial amount of the disordered RS phase (close to 30 wt %) formed as well (Figure S5). As the cation composition of this RS phase is unknown, we call it ”RS (Mg,W)­N,” although Rietveld analysis shows that Mg_0.5_W_0.5_N fits the PXRD data reasonably well. Refined parameters for these two phases are shown in Tables S2 and S3. Both nitride phases are stable in air (Figure S9).

*In situ* synchrotron PXRD shows that the reaction between 3MgCl_2_ and Li_6_WN_4_ has a small temperature window between 380 and 440 °C for the selective synthesis of WZ Mg_3_WN_4_ over RS (Mg,W)N (Figure 2). Reactivity is first observed at 280 °C, as the precursor peaks decrease in intensity and (Li,Mg)­Cl peaks appear. By 380 °C, WZ Mg_3_WN_4_ peaks appear but are weak and grow slowly. Near 440 °C, peaks consistent with RS (Mg,W)N grow. In the 410–510 °C range, MgCl_2_ peaks fade out since it is consumed for nitride formation and the reaction with LiCl: 2LiCl + MgCl_2_ → Li_2_MgCl_4_. At 510 °C, the spinel Li_2_MgCl_4_ phase converges with the RS (Li,Mg)Cl phase. These changes are consistent with the LiCl-MgCl_2_ phase diagram.^25^ Slightly before the (Li,Mg)Cl chloride melts (540 °C), the Li_6_WN_4_ precursor is completely consumed and both sets of nitride peaks grow rapidly. By 580 °C, WZ Mg_3_WN_4_ and RS (Mg,W)N are the only crystalline phases present (in a flux of liquid LiCl), and no further reaction is observed. No other peaks appear or disappear during the 30 min dwell, nor do the nitride peaks change in intensity.

Following *in situ* PXRD, we attempted several synthetic conditions *ex situ* to isolate phase-pure WZ Mg_3_WN_4_. These experimental results are shown by the synthesis map for the 3MgCl_2_ + Li_6_WN_4_ mixture with the dwell time and temperature as the map coordinates (Figure 3a). Because all PXRD data for the map were collected and analyzed for unwashed samples, we did not estimate the ratio of products. For clarity, only W-containing phases are shown in Figure 3, since every unwashed product also contains LiCl salts. At a low dwell time (<12 h), all our *ex situ* experimental results align well with the *in situ* synchrotron data, shown as a dotted line in Figure 3a. The syntheses at ≤ 500 °C showed residual unreacted Li_6_WN_4_ (Figure S6), while prolonged heating (>24 h) at ≥500 °C leads to complete consumption of Li_6_WN_4_. Interestingly, heating at 600 °C for up to 150 h did not change the WZ Mg_3_WN_4_ structure (Figure S7). This result indicates that even though WZ is enthalpically metastable at 0 K,^10^ it is stable enough to withstand 600 °C for 150 h, which is encouraging for practical applications of this material. Even as low as 600 °C, trace amounts of MgWN_2_ (less than 1 wt % is visible only for the washed sample) appear in PXRD after heating for 12 h (Figure S5). At 800 °C and above, the MgWN_2_ rockseline (RL) structure can be detected (Figure S6), indicating decomposition via Mg species gas phase loss. (less than 1 wt % is visible only for the washed sample) appear in PXRD after heating for 12 h (Figure S5).

**Figure 3:**
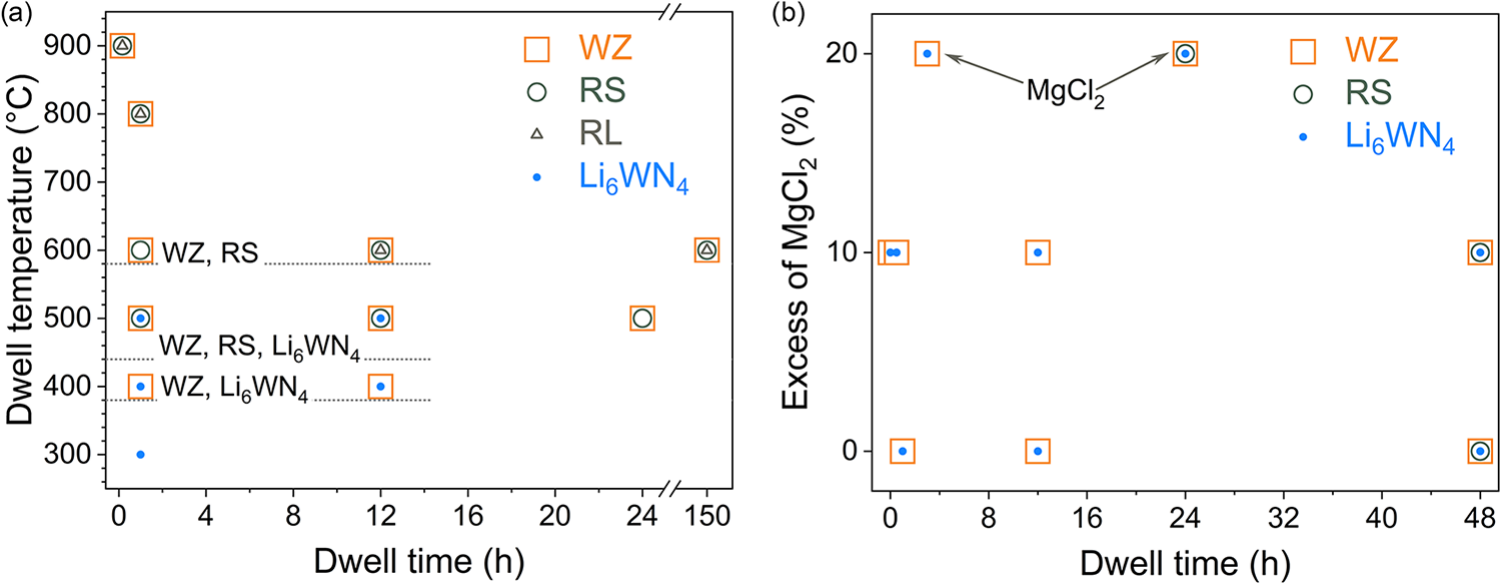
Synthesis map for the 3MgCl_2_ + Li_6_WN_4_ mixture (a) and the (3+*x*)­MgCl_2_ + Li_6_WN_4_ mixture at 400 °C (b). Symbols represent *ex situ* data and dotted lines correspond to the temperatures of phase transformations from *in situ* measurements.

Since the RS phase formation was observed in *in situ* studies above 440 °C, we explored the synthesis map for the MgCl_2_−Li_6_WN_4_ system more thoroughly at 400 °C (Figure 3b). The increased dwell time at 400 °C led to the appearance of the RS (Mg, W)N phase, whose crystallinity improved with increased reaction time (24 vs 48 h, Figure S10). One of the possible reasons for the challenges associated with WZ Mg_3_WN_4_ phase purity was that the formation of (Li,Mg)Cl and Li_2_MgCl_4_ creates Mg-poor reaction media.^26,27^ However, adding excess MgCl_2_ to the reaction at 10% and 20% did not affect the reaction outcome, i.e., unreacted Li_6_WN_4_ (Figures 3 and S11). More interestingly, there is a noticeable amount of unreacted MgCl_2_ salt present for the 20% excess MgCl_2_ reactions (Figure S11). Therefore, to eliminate the Li_6_WN_4_ impurity, we employed methanol washing to dissolve/decompose the side product, leaving only WZ Mg_3_WN_4_ as the final powder (Figure 4). The purest WZ Mg_3_WN_4_ was obtained in the reaction with 10% excess MgCl_2_, heated at 400 °C for 0.5 h.

**Figure 4:**
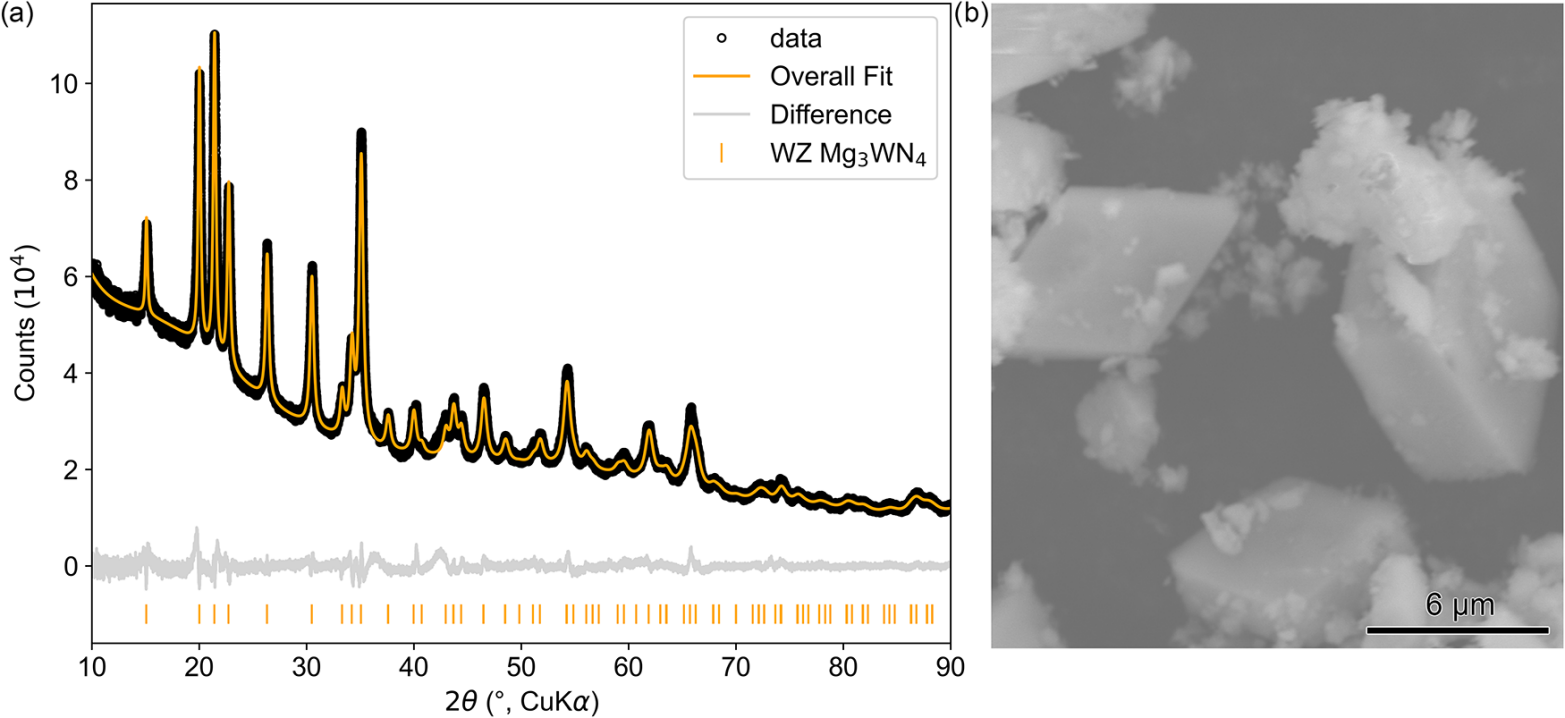
Refinement of ex situ laboratory PXRD data (a) and SEM image (b) of WZ Mg_3_WN_4_ synthesized at 400 °C for 0.5 h (left).

**Table 1:** Atomic Coordinates for WZ Mg_3_WN_4_ Synthesized at 400 °C for 0.5 h from Rietveld Refinements of Laboratory PXRD Data in the Orthorhombic Space Group *Pmn*2_1_ (no. 31)^a^

site	element	mult.	*x*	*y*	*z*	occupancy	*U*_iso_
Mg1	Mg	4	0.23742(26)	0.1457(7)	0.2027(11)	1	0.00559(8)
Mg2	Mg	2	0	0.3423(9)	0.710(3)	1	0.00559(8)
W1	W	2	0	0.66910(13)	0.2098(5)	1	0.00559(8)
N1	N	4	0.2477(6)	0.8126(12)	0.0638(6)	1	0.01
N2	N	2	0	0.3894(12)	0.1264(12)	1	0.01
N3	N	2	0	0.7009(14)	0.5517(11)	1	0.01

a Cell parameters refined to *a* = 6.75051(16), *b* = 5.85364(13), and *c* = 5.23247(10) Å. The sample was obtained by heating at 400 °C for 0.5 h.

Rietveld refinement of the WZ Mg_3_WN_4_ synthesized via heating at 400 °C for 0.5 h resulted in a very similar structure to that obtained during initial synthesis at 600 °C (Figures S5 and 4a and Tables 1 and S2). The main difference between these structures is that WZ Mg_3_WN_4_ synthesized at 400 °C does not exhibit antisite mixing as observed at 600 °C. The WZ-derived Mg_3_WN_4_ structure obtained at 600 °C has cation mixing on the Mg sites with the mixing level ranging at 3−4% (Table S2). At the same time, the cation-disordered WZ refinement model is not preferential for Mg_3_WN_4_ synthesized at 400 °C. Free refinement suggests electron deficiency on the Mg_2_ site, which cannot be modeled with the heavier W atom. There is also electron abundance on the Mg1 site that can be modeled with W mixing, but this resulted in much less than 1% disorder, which is insignificant.

SEM images of the WZ Mg_3_WN_4_ synthesized via heating at 400 °C for 0.5 h demonstrated diamond-shaped 5 × 5 × 2 μm^3^ single-crystal particles with some amorphous material covering their surface (Figure 4b). In an optical microscope, these particles appeared to be transparent colorless crystallites covered with amorphous black powder (Figure 5a inset). EDS mapping showed homogeneous Mg and W distribution for both particles and the amorphous material (Figure S13). Estimating the Mg/W ratio for particles resulted in 2:1 (areas 1 and 2 in Figure S16), suggesting the material composition LiMg_2_WN_4–δ_ with the WZ-derived crystal structure, which has not been theoretically or experimentally reported to date. For the amorphous material, the Mg/W ratio was higher (2.6:1) with increased chlorine content, which might be the salt residue or a small fraction of some other amorphous material undetected by XRD (Figure S14 and Table S5). As we increased the reaction time from 0.5 to 12 h at 400 °C, we noticed the disintegration of the diamond-shape crystallites (area 1 in Figure S15) and the formation of agglomerates consisting of smaller particles (areas 2 and 3 in Figure S15). EDS analysis of these three areas resulted in 2.8:1 Mg/W ratio, suggesting that more Mg intercalation in the WZ Mg_3_WN_4_ structure destroys the diamond-shaped morphology, also altering the color of the sample toward gray or black.

**Figure 5:**
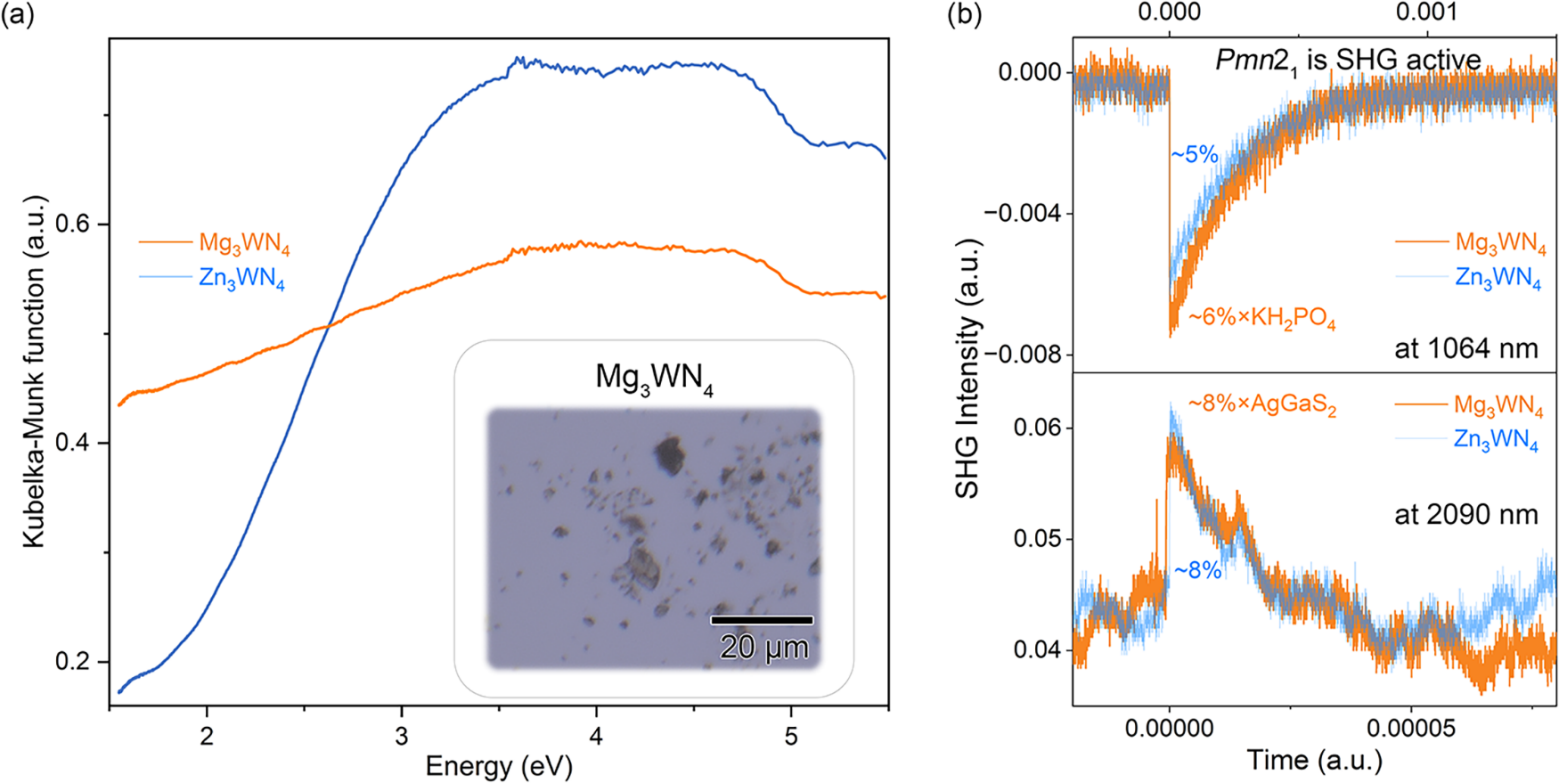
Absorbance spectra (a) and SHG activity (b) of WZ Mg_3_WN_4_ and Zn_3_WN_4_. The inset shows the optical image of the WZ Mg_3_WN_4_ sample.

Similarly, WZ Mg_3_WN_4_ powder prepared at 600 °C for 12 h resulted in even more substantial agglomerates forming (Figure S16). In this case, diamond-shaped WZ Mg_3_WN_4_ particles with 3.1:1 Mg/W ratio are barely preserved (area 1 in Figure S16). Even though these EDS results are quantitative and not qualitative, due to the large electron difference between Mg and W affecting results, the measured differences suggest that there is some Mg/W off-stoichiometry ranging between 2 and 3 that can be stabilized in the WZ Mg_3_WN_4_ structure at various synthetic conditions. A smaller Mg/W ratio can be tentatively explained with Li or O presence in the WZ Mg_3_WN_4_ structure, while a higher Mg/W ratio might be associated with Mg substitution on the W site.

We collected diffuse reflectance spectra for the WZ Mg_3_WN_4_ sample and its Zn analog.^5^ While WZ Zn_3_WN_4_ has a well-defined optical absorption onset above 2 eV, WZ Mg_3_WN_4_ exhibits a much slower onset with absorption slowly increasing from <1.5 eV to >4 eV (the data collection limit, Figure 5a). It is likely that this optical absorption in the visible region of the spectrum corresponds to the defective WZ structure masking the intrinsic band gap of WZ Mg_3_WN_4_. Defects like Mg/W antisite disorder and Li or O substitution in the structure can affect the density of states between the conduction and valence bands, resulting in gray or even black color, even though WZ Mg_3_WN_4_ is predicted to be a wide-band gap semiconductor with expected yellow or white color. Therefore, defects in the WZ Mg_3_WN_4_ structure play a crucial role in material properties and should be understood and mitigated before the characterization of ferroelectricity or Mg^2+^ ion conductivity in this material.

Nevertheless, to probe the material properties related to ferroelectric applications, we performed second-harmonic generation (SHG) measurement for WZ Mg_3_WN_4_ reported here, as well as for its Zn-analog—WZ Zn_3_WN_4_.^5^ The SHG intensities of Mg_3_WN_4_ and Zn_3_WN_4_ were found to be approximately 0.08 times that of AgGaS_2_ at 2090 nm (Figure 5b). At 1064 nm, the SHG responses of WZ Mg_3_WN_4_ and Zn_3_WN_4_ correspond to approximately 0.06 and 0.05 times that of KH_2_PO_4_, respectively (Figure 5b). Although the observed SHG signals are relatively weak, these results provide clear evidence for the noncentrosymmetric structure of WZ Mg_3_WN_4_ and Zn_3_WN_4_ materials and support their crystallization in a polar *Pmn*2_1_ space group determined from XRD.

## Conclusions

This paper details the successful synthesis of the cation-ordered wurtzite structure of Mg_3_WN_4_ via a metathesis reaction, 3MgCl_2_ + Li_6_WN_4_. *In situ* synchrotron PXRD shows that a main contaminant, RS (Mg,W)­N, begins forming at a slightly higher temperature than that of WZ Mg_3_WN_4_ (440 °C vs 380 °C). Narrowing the temperature window to 400 °C for *ex situ* WZ material synthesis for 0.5 h with 10% excess resulted in deriving the phase-pure WZ Mg_3_WN_4_. *Ex situ* PXRD corroborated by SHG measurements shows that this phase crystallizes in the polar *Pmn*2_1_ space group with Mg^2+^ in a tetrahedral coordination environment. The combination of Rietveld refinements probing the Mg and W site occupancies, EDS spectroscopy analysis of the Mg/W ratio for separate particles, and optical measurements of bulk WZ Mg_3_WN_4_ powder suggested complex defect chemistry in WZ Mg_3_WN_4_. Overall, the results presented in this paper demonstrate the *ex situ* synthesizability of the WZ Mg_3_WN_4_ informed by *in situ* synchrotron PXRD and pave a way toward the future property measurements that would assess the viability of this unusual material as a ferroelectric or Mg^2+^ ion conductor.

## Supplementary Material



## Notes

Additional *in situ* XRD analysis, *ex situ* PXRD experiments on Mg_3_WN_4_, compositional analysis by EDS and SEM micrograph, ion exchange thermodynamics, and salt melting points (PDF)

## References

[1] Rong Z., Malik R., Canepa P., Sai Gautam G., Liu M., Jain A., Persson K., Ceder G. (2015). Materials design rules
for multivalent ion mobility in intercalation structures. Chem. Mater..

[2] Canepa P., Bo S.-H., Sai Gautam G., Key B., Richards W. D., Shi T., Tian Y., Wang Y., Li J., Ceder G. (2017). High magnesium
mobility in ternary spinel chalcogenides. Nat.
Commun..

[3] Lee C.-W., Din N. U., Yazawa K., Brennecka G. L., Zakutayev A., Gorai P. (2024). Emerging materials
and design principles
for wurtzite-type ferroelectrics. Matter.

[4] Fichtner S., Wolff N., Lofink F., Kienle L., Wagner B. (2019). AlScN: AIII-V
semiconductor based ferroelectric. J. Appl.
Phys..

[5] Rom C. L., O’Donnell S., Huang K., Klein R. A., Kramer M. J., Smaha R. W., Zakutayev A. (2024). Low-temperature synthesis of cation-ordered
bulk Zn_3_WN_4_ semiconductor via heterovalent solid-state
metathesis. Chem. Sci..

[6] Sun W., Bartel C. J., Arca E., Bauers S. R., Matthews B., Orvañanos B., Chen B.-R., Toney M. F., Schelhas L. T., Tumas W., Tate J., Zakutayev A., Lany S., Holder A. M., Ceder G. (2019). A map of the inorganic
ternary metal nitrides. Nat. Mater..

[7] Stevanović V., Lany S., Zhang X., Zunger A. (2012). Correcting density
functional theory for accurate predictions of compound enthalpies
of formation: Fitted elemental-phase reference energies. Phys. Rev. B.

[8] Lany S. (2013). Band-structure
calculations for the 3 d transition metal oxides in GW. Phys. Rev. B.

[9] Lany S. (2015). Semiconducting
transition metal oxides. J. Phys.: Condens.
Matter.

[10] Rom C. L., Smaha R. W., Knebel C. A., Heinselman K. N., Neilson J. R., Bauers S. R., Zakutayev A. (2023). Bulk and film
synthesis pathways to ternary magnesium tungsten nitrides. J. Mater. Chem. C.

[11] Zakutayev A., Jankousky M., Wolf L., Feng Y., Rom C. L., Bauers S. R., Borkiewicz O., LaVan D. A., Smaha R. W., Stevanovic V. (2024). Synthesis pathways to thin films of stable layered
nitrides. Nat. Synth..

[12] Rom C. L., Jankousky M., Phan M. Q., O’Donnell S., Regier C. E., Neilson J. R., Stevanovic V., Zakutayev A. (2025). Ion Exchange Synthesizes a Metastable
Layered Polymorph
of MgZrN_2_ and MgHfN_2_ Semiconductors. Chem. Mater..

[13] Rom C. L., Fallon M. J., Wustrow A., Prieto A. L., Neilson J. R. (2021). Bulk Synthesis,
Structure, and Electronic Properties of Magnesium Zirconium Nitride
Solid Solutions. Chem. Mater..

[14] Todd P. K., Fallon M. J., Neilson J. R., Zakutayev A. (2021). Two-step solid-state
synthesis of ternary nitride materials. ACS
Mater. Lett..

[15] Bauers S. R., Holder A., Sun W., Melamed C. L., Woods-Robinson R., Mangum J., Perkins J., Tumas W., Gorman B., Tamboli A., Ceder G., Lany S., Zakutayev A. (2019). Ternary nitride
semiconductors in the rocksalt crystal structure. Proc. Natl. Acad. Sci. U.S.A..

[16] Yang M., Zakutayev A., Vidal J., Zhang X., Ginley D. S., DiSalvo F. J. (2013). Strong optical absorption in CuTaN_2_ nitride
delafossite. Energy Environ. Sci..

[17] Zakutayev A., Allen A. J., Zhang X., Vidal J., Cui Z., Lany S., Yang M., DiSalvo F. J., Ginley D. S. (2014). Experimental
synthesis and properties of metastable CuNbN_2_ and theoretical
extension to other ternary copper nitrides. Chem. Mater..

[18] Yuan W. X., Hu J., Song Y., Wang W., Xu Y. (2005). Synthesis and structure
of the ternary nitride Li_6_WN_4_. Powder Diffr..

[19] Coelho A. A. (2018). TOPAS and
TOPAS-Academic: an optimization program integrating computer algebra
and crystallographic objects written in C++. J. Appl. Crystallogr..

[20] Toby B. H., Von Dreele R. B. (2013). GSAS-II: The Genesis of a Modern
Open-Source All Purpose
Crystallography Software Package. J. Appl. Crystallogr..

[21] Chupas P. J., Chapman K. W., Kurtz C., Hanson J. C., Lee P. L., Grey C. P. (2008). A versatile sample-environment
cell for non-ambient
X-ray scattering experiments. J. Appl. Crystallogr..

[22] Todd P. K., Smith A. M., Neilson J. R. (2019). Yttrium
manganese oxide phase stability
and selectivity using lithium carbonate assisted metathesis reactions. Inorg. Chem..

[23] Kurtz S. K., Perry T. (1968). A powder technique for the evaluation
of nonlinear optical materials. J. Appl. Phys..

[24] Breternitz J., Schorr S. (2021). Symmetry relations
in wurtzite nitrides and oxide nitrides
and the curious case of *Pmc*2_1_. Acta Crystallogr., Sect. A.

[25] Lutz H. D., Schmidt W., Haeuseler H. (1979). Zur Kenntnis
der Chlorid-Spinelle
Li_2_MgCl_4_, Li_2_MnCl_4_, Li_2_FeCl_4_, Li_2_CdCl_4_. Z. Anorg. Allg. Chem..

[26] Wustrow A., Key B., Phillips P. J., Sa N., Lipton A. S., Klie R. F., Vaughey J. T., Poeppelmeier K. R. (2018). Synthesis
and Characterization of
MgCr_2_S_4_ Thiospinel as a Potential Magnesium
Cathode. Inorg. Chem..

[27] Rom C. L., Novick A., McDermott M. J., Yakovenko A. A., Gallawa J. R., Tran G. T., Asebiah D. C., Storck E. N., McBride B. C., Miller R. C., Prieto A. L., Persson K. A., Toberer E., Stevanović V., Zakutayev A., Neilson J. R. (2024). Mechanistically Guided Materials
Chemistry: Synthesis
of Ternary Nitrides, CaZrN_2_ and CaHfN_2_. J. Am. Chem. Soc..

