# Supplementary material for: Synthesis and Properties of Bulk Mg3WN4 in a Wurtzite-Derived Structure
Source: Chem Mater. 2026 Jun 24;38(13):6339–46. doi: 10.1021/acs.chemmater.6c00200 (PMC13373925; doi:10.1021/acs.chemmater.6c00200)
Supplement: Supplementary file 1 [file cm6c00200_si_001.pdf]

Supporting Information for: Synthesis and properties of bulk Mg_3WN_4 in a wurtzite-derived structure

Anna A. Berseneva,[†,‖] Christopher L. Rom,[†,‖] Layton Rudolph,[‡,‖] Yunseung Kuk,[¶] P. Shiv Halasyamani,[¶] Rebecca W. Smaha,[†] James R. Neilson,[§] and Andriy Zakutayev[*,†]

†*Materials Science Center, National Laboratory of the Rockies, Golden, CO, 80401, USA*

‡*Department of Chemistry, Colorado State University, Fort Collins, Colorado 80523, USA*

¶*Department of Chemistry, University of Houston, Houston, Texas 77204, USA*

§*Department of Chemistry and School of Materials Science & Engineering, Colorado State University, Fort Collins, Colorado 80523, USA*

‖*Contributed equally to this work*

E-mail: Andriy.Zakutayev@nlr.gov

In situ Synchrotron PXRD analysis

The presence of a reflection near Q = 1.03 Å^{-1} supports the formation of spinel Li_2MgCl_4 (Figure S1). Fitting this phase was attempted; however, substantial overlap between spinel Li_2MgCl_4 and RS (Li,Mg)Cl peaks (not shown in Figure S1) and the need for refinement of the lattice parameters of the phases during heating inhibited a stable and quantitative Rietveld analysis of the Li_2MgCl_4 spinel. Therefore, we represent the Li_2MgCl_4 phase in Figure 2 using the normalized peak intensity at Q = 1.026191 Å^{-1} as a proxy for the weighted scale factor (WSF) of Li_2MgCl_4 while restricting the (Li,Mg)Cl ability to fit this peak. If we replot in situ data not as weighted scale

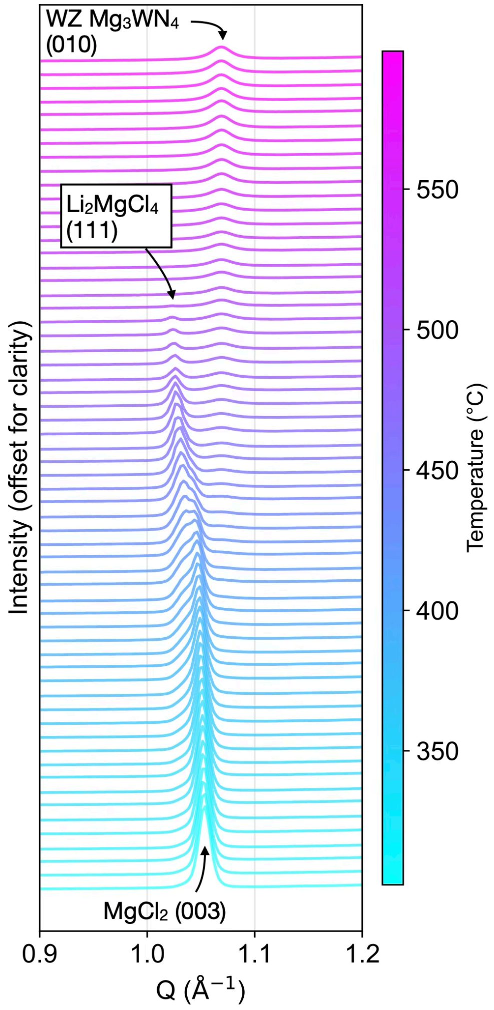

Figure S1: *In situ* synchrotron PXRD of the ramp from 300 °C to 600 °C focused on low-angle reflections, highlighting the emergence and disappearance of the (111) Li_2MgCl_4 spinel peak.

factor but mole scale factor (Figures S2 and S3), we could see how in the 300-500 °C temperature region, the refining model overestimate LiCl phase, i.e., Li, Cl elements and underestimate Mg elements. This is indictive of Mg intercalation in LiCl as well as formation of Li_2MgCl_4 which we do not fit perfectly.

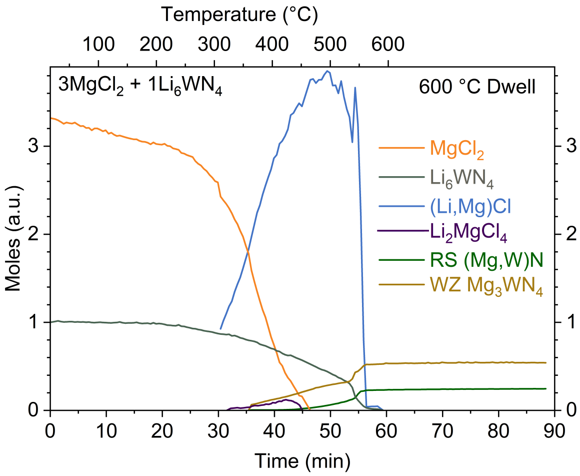

Figure S2: *In situ* synchrotron PXRD data plotted as a mole scale factor for studied phases vs time.

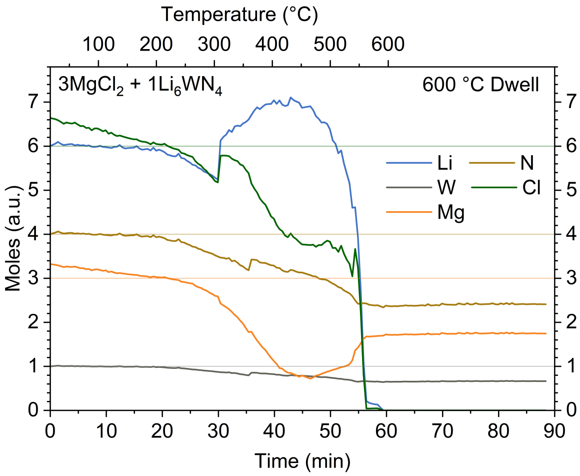

Figure S3: *In situ* synchrotron PXRD data plotted as a mole scale factor for elements vs time.

Ex situ Laboratory PXRD analysis

Ex situ PXRD patterns of reactions heated to various dwell temperatures for 1 h are shown in Figure S6. At 400 °C, peaks from the Li_6WN_4 precursor are still visible, but the $MgCl_2$ peaks are not present indicating that some reactivity has occurred. However, peaks for the expected LiCl and Mg_3WN_4 WZ products do not appear until after heating at 500 °C, although trace Li_6WN_4 persists. By 600 °C, the precursors are fully consumed and Mg_3WN_4 is crystallized, although this pattern shows a large unidentified reflection near 33° potentially Li_3OCl. These patterns are of insufficient quality to resolve the RS (Mg,W)N phase, although it is likely present given the *in situ* PXRD observations (Figure 2). The 800 °C reaction shows evidence of RL $MgWN_2$ in addition to WZ Mg_3WN_4. This suggests that the metastable WZ Mg_3WN_4 material exhibits modest thermal stability even at high temperatures that begin driving conversion to the thermodynamic ground state (RL $MgWN_2$). Extended heating at 600 °C does not seem to convert the WZ Mg_3WN_4 phase into the RL polymorph (Figure S7). Note: the Kapton tape, the polyimide film we used to protect sample from interaction with air and moisture, has a strong background peak that hides phases below 30 deg, therefore phases with up to 2 wt. % could not be captured.

Washing with anhydrous methanol successfully removes LiCl (Figure S8). After removing the polyimide film, the WZ Mg_3WN_4, RS (Mg,W)N, and $MgWN_2$ peaks become substantially more visible. Rietveld refinement was performed for the sample obtained after 12-hours heating at 600 °C followed by methanol washing. The lattice parameters, atom coordinates, site disorder, crystal size broadening, and thermal parameters were refined for the WZ Mg_3WN_4 phase ($Pmn2_1$, R_{wp} = 4.737 %.) and presented in S2. Site disorder for all Mg and W sites was constrained to result in 1 total occupancy for each site. Disorder on the metal sites improved the fit substantially (from a R_{wp} of 5.169 to a R_{wp} of 4.737 with adding 3 more refinable parameters) and passed a significance level of 0.10.[1] The thermal parameters for all atoms were refined together with an equivalence constraint. A Lorentzian size broadening factor was used to refine the broadness of the peak, resulting in a crystal size of 21.4 nm. The nitrogen occupancy and location were kept fixed because allowing them to refine did not pass a significance level of 0.10 (starting from R_{wp} of 4.737 to a

value of 4.363 with 7 refinable parameters).[1] The nitrogen atom coordinates were taken from the Zn_3WN_4 $Pmn2_1$ structure from Materials Project.[2] The RS (Mg,W)N phases lattice parameters, Lorentzian crystal size broadening, and Lorentzian strain broadening were refined. The structure's original Mg:W ratio of 0.5:0.5 on the metal site was held constant. When occupancies were refined freely, the occupancies stayed the same; therefore, we fixed them at 1-to-1 ratio. The crystal size refined to a value of 51.5 nm. The $MgWN_2$ phase had very little intensity in the washed impure sample and only makes up less than one weight percent of the sample, making further analysis of this phase difficult. These phases did not show signs of degradation in air (Figure S9). The PXRD pattern remains the same after 3 hours of sequential measurements.

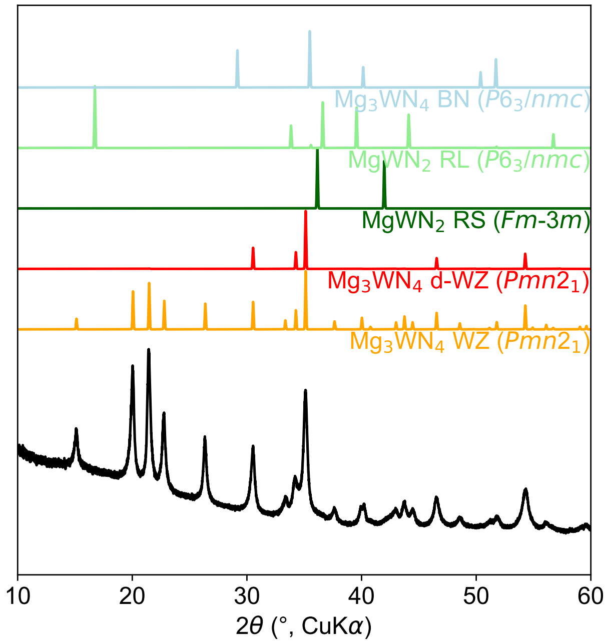

Figure S4: Laboratory PXRD pattern of WZ Mg_3WN_4 (bottom) in comparison with other structures in the Mg-W-N chemical space (top). d-WZ Mg_3WN_4 and WZ Mg_3WN_4 patterns show cation-disordered and cation-ordered structures, respectively.

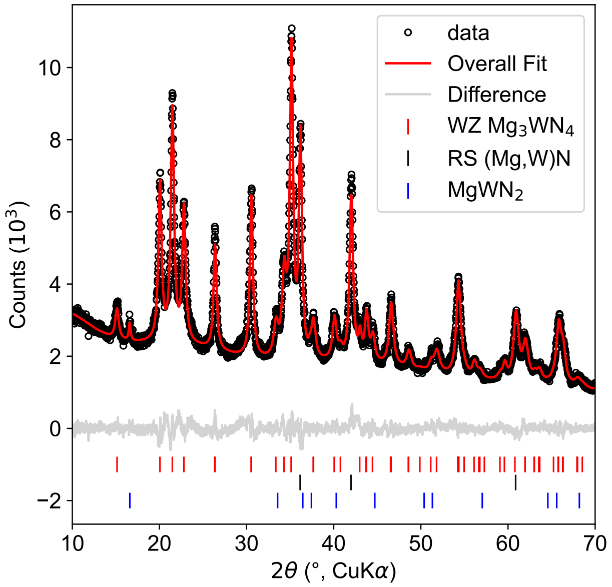

Figure S5: Ex situ laboratory PXRD data shows WZ Mg_3WN_4 (72 wt. %), RS (Mg,W)N (27 wt. %), and RL $MgWN_2$ (1 wt. %) are the products of a reaction between $3MgCl_2 + Li_6WN_4$ (heated at 600 °C for 12 h, then washed with anhydrous methanol).

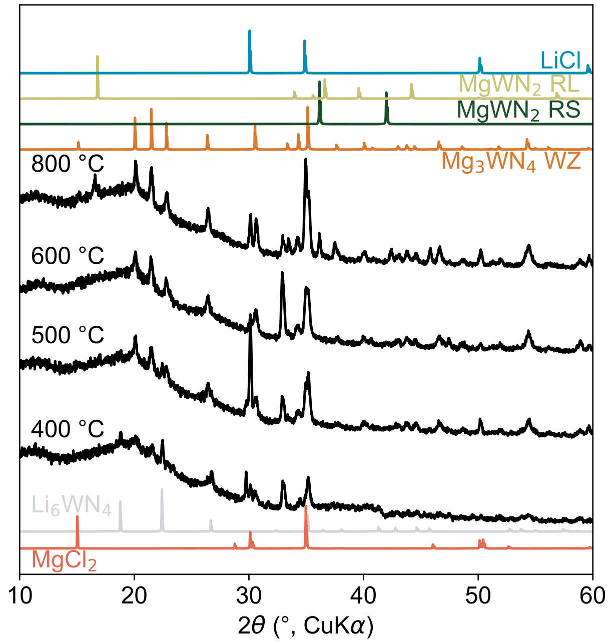

Figure S6: Laboratory PXRD patterns for reactions between $Li_6WN_4 + 3MgCl_2$ heated to various temperatures for 1 h dwell times. Simulated reference patterns are shown for precursors (bottom) and products (top). The large background at low angle stems from the polyimide tape used to protect the sample from moisture during the measurement.

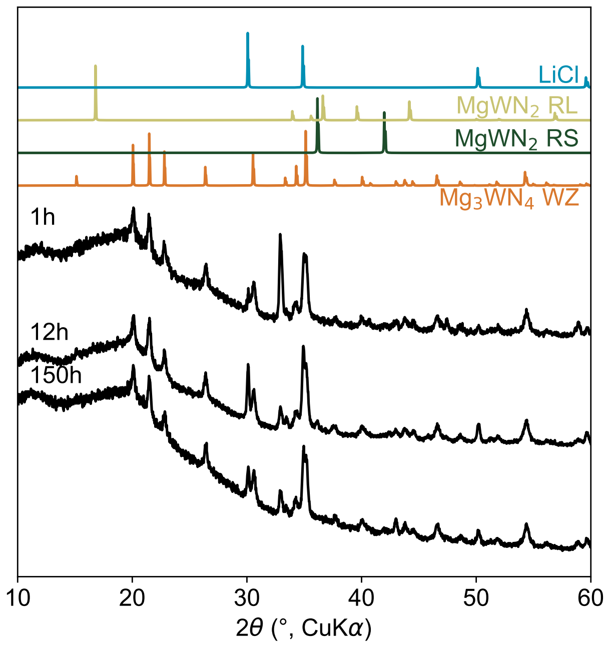

Figure S7: Laboratory PXRD patterns for reactions between $Li_6WN_4 + 3MgCl_2$ heated at 600 °C for various dwell times. Simulated patterns for possible products are shown for reference (top). The large background at low angle stems from the polyimide tape used to protect the sample from moisture during the measurement. Identification of the RL phase is masked by the Kapton tape at few weight percentage.

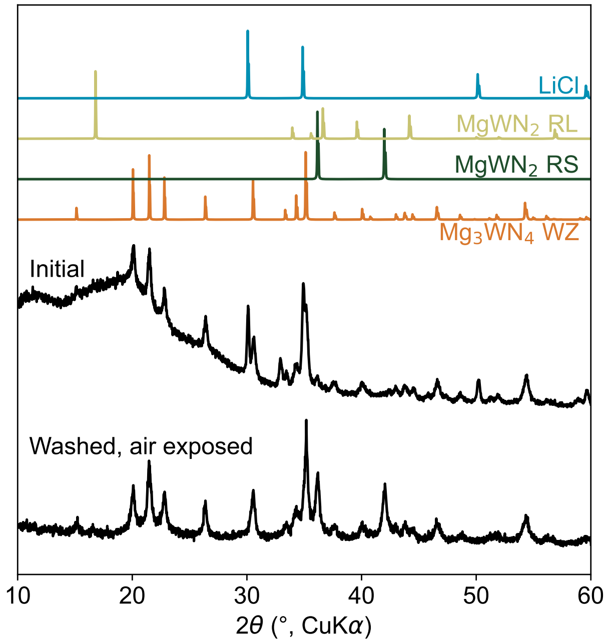

Figure S8: Laboratory PXRD data of initial and washed sample after reaction between between $Li_6WN_4 + 3MgCl_2$ showing that washing the product with anhydrous methanol removes LiCl but leaves the WZ Mg_3WN_4 and RS (Mg,W)N phases intact.

Table S1: Data collection parameters for WZ Mg_3WN_4.

Synthesis conditions	600°C-12h	400°C-0.5h
Empirical formula	Mg_3WN_4	
Formula weight	312.79	
Temperature	rt	
Crystal system	orthorhombic	
Space group	$Pmn2_1$	
a, Å	6.7566(7)	6.76051(16)
b, Å	5.8522(7)	5.85364(13)
c, Å	5.2245(5)	5.23247(10)
α,°	90	
β, °	90	
γ, °	90	
Volume, $Å^3$	206.58(4)	207.068(8)
Z	2	
ρ_{calc}, g/cm^3	5.2596	5.0168
Radiation	$CuK\alpha_1/K\alpha_2$ (1/2)	
Instrument	Rigaku Ultima IV	Rigaku SmartLab
2θ range for data collection, °	10 to 70	10 to 90
Reflections collected	58	106
Goodness-of-fit	2.45	4.86
R_{wp}, %	4.745	2.869

Table S2: Refined atomic coordinates for WZ Mg_3WN_4 from Rietveld refinements of laboratory PXRD data in orthorhombic space group $Pmn2_1$ (no. 31). Cell parameters refined to $a = 6.7566(7)$, $b = 5.8522(7)$, and $c = 5.2245(5)$ Å. The sample was obtained by heating at 600 °C for 12 hours.

site	element	mult.	x	y	z	occupancy	B_{iso}
Mg1	Mg	4	0.2339(11)	0.165(4)	0.190(4)	0.960(5)	1.31(9)
Mg1	W	4	0.2339(11)	0.165(4)	0.190(4)	0.040(5)	1.31(9)
Mg2	Mg	2	0	0.311(5)	0.762(5)	0.967(6)	1.31(9)
Mg2	W	2	0	0.311(5)	0.762(5)	0.033(6)	1.31(9)
W1	W	2	0	0.6777(9)	0.224(3)	0.99(3)	1.31(9)
W1	Mg	2	0	0.6777(9)	0.224(3)	0.01(3)	1.31(9)
N1	N	4	0.23710	0.82682	0.11320	1	1.31(9)
N2	N	2	0	0.35650	0.11271	1	1.31(9)
N3	N	2	0	0.67108	0.57976	1	1.31(9)

Table S3: Refined atomic coordinates for RS (Mg,W)N from Rietveld refinements of laboratory PXRD data in cubic space group $Fm\bar{3}m$ (no. 225). Cell parameter refined to $a = 4.3006(2)$ Å. The sample was obtained by heating at 600 °C for 12 hours.

Site	x	y	z	occ.	B_{iso}
Mg	0	0	0	0.5	0.6
W	0	0	0	0.5	0.6
N	0.5	0.5	0.5	0.5	0.4

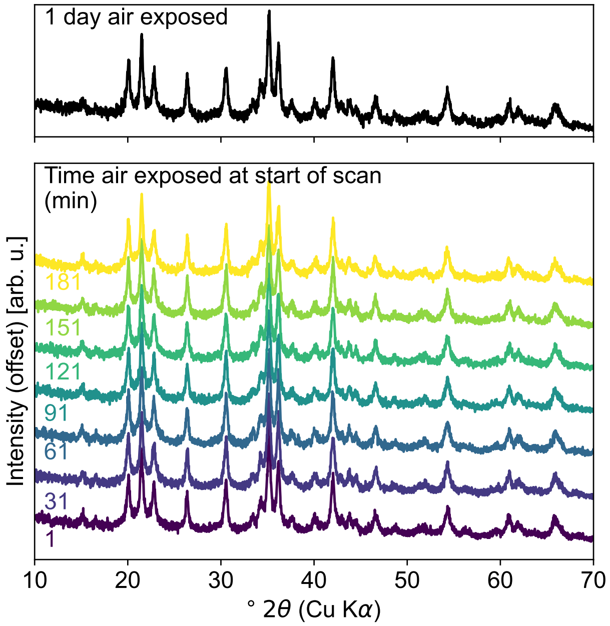

Figure S9: Laboratory PXRD data show that Mg_3WN_4 is stable in air for at least 1 day.

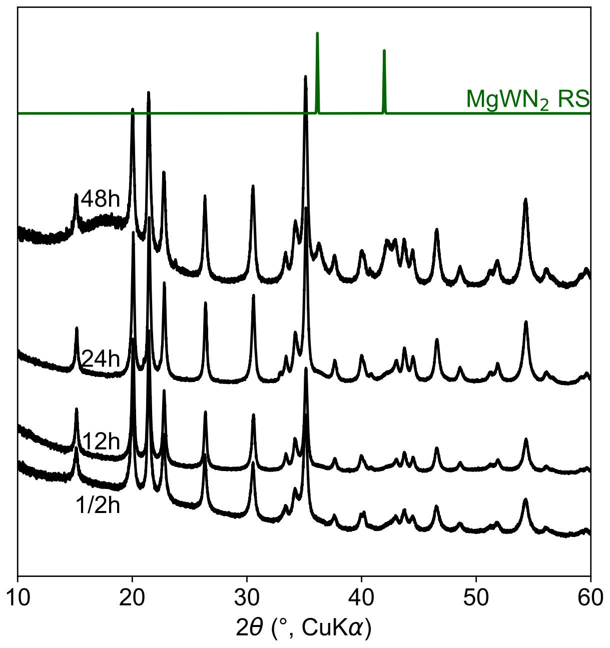

Figure S10: Laboratory PXRD pattern of washed WZ Mg_3WN_4 synthesized at 400 °C with different dwell times (bottom) in comparison with $MgWN_2$ (top). The large background at low angle for 48 h sample stems from the Vaseline we used to secure sample on the zero-background slide, but since it showed such a big background, we stopped using it.

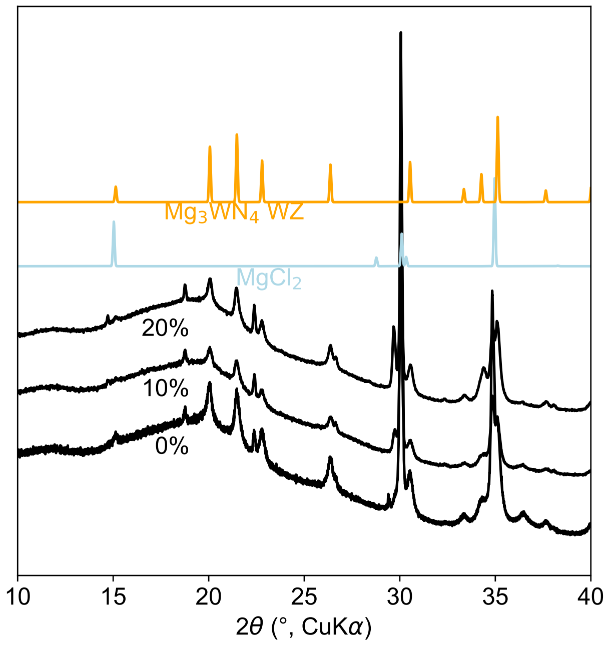

Figure S11: Laboratory PXRD pattern of unwashed WZ Mg_3WN_4 synthesized with various excess of $MgCl_2$ salt (bottom) in comparison with $MgCl_2$ and WZ Mg_3WN_4 (top). The large background at low angle stems from the polyimide tape used to protect the sample from moisture during the measurement.

Compositional analysis

Energy dispersive X-ray spectroscopy (EDS) quantifies atomic percentages as shown in Tables S4 and S5. These data are derived from the SEM image and EDS spectra shown in Figures S12, S14, S15, and S16. These measurements show the presence of nitrogen, although the derived values should be treated semi-quantitatively owing to the low energy of the emissions.

For the sample synthesized by 12-hour reaction at 600 °C we estimated the bulk elemental composition by using areas covering agglomerates and not single particles for EDS data collection. The average Mg/W ratio is 3.83 (Table S4), which is higher than the 3.0 expected for Mg_3WN_4. This likely indicates that the RS (Mg,W)N phase is Mg-rich, or it may stem from Mg-based contamination not detected by XRD.

Additionally, wavelength dispersive X-ray fluorescence (WD-XRF) was utilized to corroborate elemental composition of the bulk sample. The Mg/W ratio yielded 3.79, which matches well the EDS results.

Table S4: Elemental analysis of Mg_3WN_4 powders from the SEM areas in Figure S12 and WD-XRF.

Atomic %	Mg	W	N	Cl	O	Mg/W
Area 1	36.7±0.2	9.0±0.4	19.2±1.0	1.1±0.1	34.1±0.6	4.07±0.17
Area 2	33.5±0.4	9.5±0.2	20.0±3.5	0.7±0.1	36.3±1.6	3.51±0.09
Area 3	36.4±0.2	9.4±0.1	19.7±1.4	1.0±0.1	33.5±0.7	3.89±0.05
WDXRF	76.84	20.27	-	2.89	-	3.79

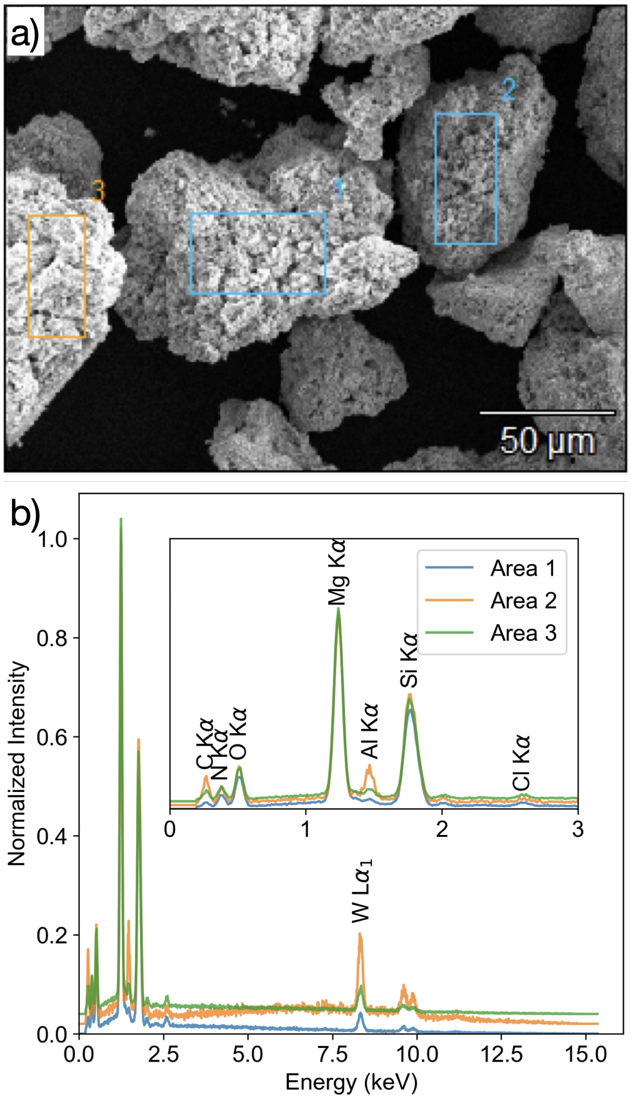

Figure S12: a) SEM image and b) EDS spectra used for quantification shown in Table S4. Inset shows details for the low-energy region. Traces offset slightly for clarity.

For the analysis of particle shape and composition, we used higher magnification during SEM measurements. In this case we were comparing elemental composition between different particle shapes presented in Figures S14, S15, and S16. Therefore, WD-XRF was not used for these samples. The Mg/W ratios for particles was below 3 (Table S5), lower than expected for Mg_3WN_4. Altogether with bulk sample analysis, this mostly points out the presence of salt residue or an undetected by XRD Mg-rich phase in the bulk sample.

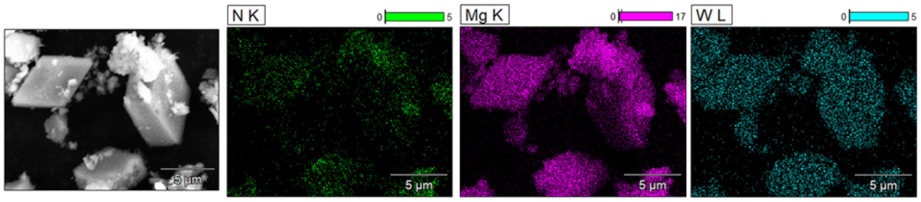

Figure S13: EDS mapping for Mg_3WN_4 powder synthesized at 400 °C for 0.5 hour.

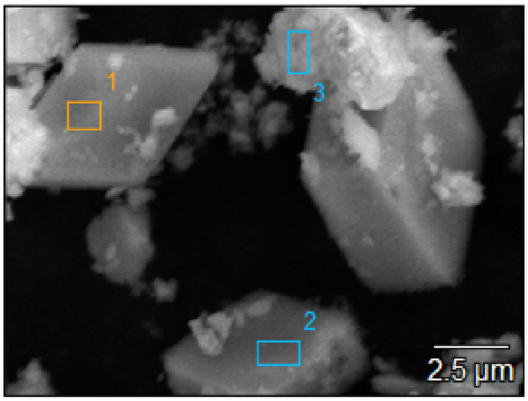

Figure S14: SEM image for Mg_3WN_4 powder synthesized at 400 °C for 0.5 hour.

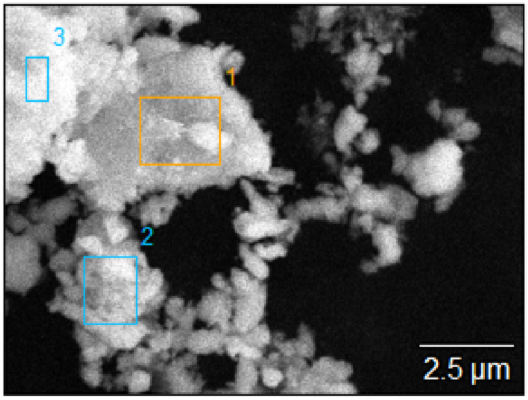

Figure S15: SEM image for Mg_3WN_4 powder synthesized at 400 °C for 12 hours.

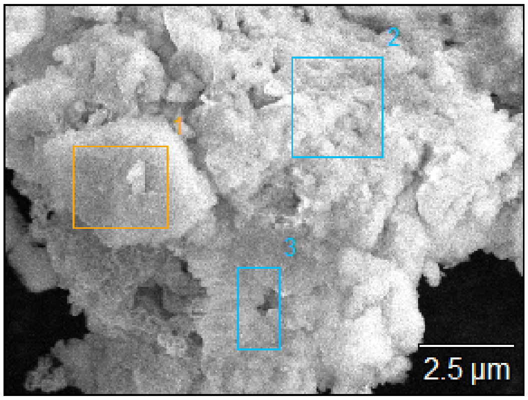

Figure S16: SEM image for Mg_3WN_4 powder synthesized at 600 °C for 12 hours.

Table S5: Elemental analysis of Mg_3WN_4 powders from the SEM areas in Figures S14, S15, and S16.

Atomic %	Mg	W	N	Cl	O	Mg/W
Mg_3WN_4 powder synthesized at 400 °C for 0.5 hour						
Area 1	17.1±0.1	8.6±0.0	7.5±1.6	0.2±0.0	70.5±0.6	1.99±0.01
Area 2	15.7±0.1	7.7±0.0	7.2±0.4	0.2±0.0	73.9±0.6	2.03±0.01
Area 3	23.2±0.21	9.1±0.0	7.4±0.9	1.2±0.1	55.9±0.7	2.56±0.01
Mg_3WN_4 powder synthesized at 400 °C for 12 hours						
Area 1	25.2±0.2	9.1±0.2	27.3±3.4	1.2±0.2	27.2±1.4	2.76±0.03
Area 2	33.7±0.3	11.7±0.0	22.8±2.4	1.6±0.0	30.2±0.9	2.89±0.03
Area 3	29.2±0.1	10.6±0.0	34.1±1.0	0.3±0.0	25.8±0.6	2.77±0.01
Mg_3WN_4 powder synthesized at 600 °C for 12 hours						
Area 1	41.4±0.3	13.5±0.1	21.6±1.4	1.2±0.0	22.4±0.8	3.08±0.02
Area 2	28.8±0.1	10.0±0.1	30.0±0.9	0.5±0.0	30.8±0.6	2.88±0.01
Area 3	26.5±0.2	7.7±0.1	21.7±3.0	0.8±0.0	73.9±1.5	2.40±0.02

Thermodynamics

As we described previously,[3] Hess's Law for reaction enthalpy can be simplified for the sake of comparing different precursors. Because Li_6WN_4 and Mg_3WN_4 are constant, we can consider the hypothetical reaction $3MgX_2 + 6Li \longrightarrow 6LiX + 3Mg$ to assess how different choices for MgX_2 may affect the exothermicity of the reaction targeting Mg_3WN_4. Figure S17 a shows that selecting MgF_2 would yield the smallest amount of heat release, which we previously showed correlated with phase purity for the target compound.[4] However, the fluorides also have the highest melting points (Figure S17b), therefore leading to slow kinetics which may inhibit selective synthesis. Although the chloride, bromide, and iodide all exhibit similar exothermicity, LiBr and LiI have lower melting points than LiCl, which may lead to more favorable kinetics at lower temperatures.

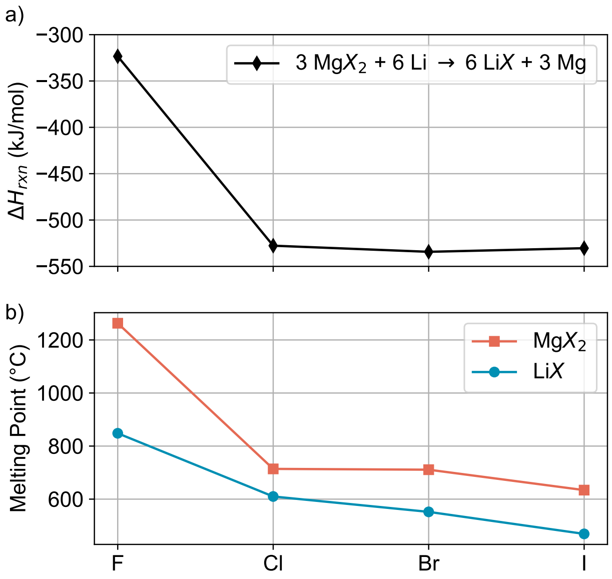

Figure S17: a) Reaction enthalpies calculated for $3MgX_2 + 6Li \longrightarrow 6LiX + 3Mg$ using ΔH_f values from the CRC Handbook of Chemistry and Physics.[5] b) Melting points of LiX and MgX_2 salts from CRC values.[6]

References

(1) Hamilton, W. C. Significance tests on the crystallographic R factor. *18*, 502–510.

(2) Jain, A.; Ong, S. P.; Hautier, G.; Chen, W.; Richards, W. D.; Dacek, S.; Cholia, S.; Gunter, D.; Skinner, D.; Ceder, G.; Persson, K. a. The Materials Project: A materials genome approach to accelerating materials innovation. *APL Materials* **2013**, *1*, 011002.

(3) Rom, C. L.; Jankousky, M.; Phan, M. Q.; O'Donnell, S.; Regier, C. E.; Neilson, J. R.; Stevanovic, V.; Zakutayev, A. Ion Exchange Synthesizes a Metastable Layered Polymorph of $MgZrN_2$ and $MgHfN_2$ Semiconductors. *Chemistry of Materials* **2025**, *37*, 2136–2144.

(4) Rom, C. L.; O'Donnell, S.; Huang, K.; Klein, R. A.; Kramer, M. J.; Smaha, R. W.; Zakutayev, A. Low-temperature synthesis of cation-ordered bulk Zn_3WN_4 semiconductor via heterovalent solid-state metathesis. *Chemical Science* **2024**, *15*, 9709–9718.

(5) Rumble, J. R. (ed.), "Standard Thermodynamic Properties of Chemical Substances" in CRC Handbook of Chemistry and Physics, 104th Edition (Internet Version). https://hbcp.chemnetbase.com/documents/09_04/09_04_0001.xhtml?dswid=6263 (accessed 2024-08-26).

(6) Rumble, J. R. (ed.), "Physical Constants of Inorganic Compounds" in CRC Handbook of Chemistry and Physics, 104th Edition (Internet Version). https://hbcp.chemnetbase.com/documents/09_04/09_04_0001.xhtml?dswid=6263 (accessed 2024-08-26).
